# Seasonality and Trend of Cellulitis, Herpes Zoster, and Varicella: A Nationwide Population-based Study

**DOI:** 10.31662/jmaj.2025-0010

**Published:** 2025-06-13

**Authors:** Hideaki Miyachi, Daisuke Sato, Sayuri Shimizu, Yaei Togawa, Kentaro Sakamaki, Kensuke Yoshimura

**Affiliations:** 1 Department of Dermatology, Chiba University Hospital, Chiba, Japan; 2 Center for Next Generation of Community Health, Chiba University Hospital, Chiba, Japan; 3 Department of Emergency Medicine, Massachusetts General Hospital, Harvard Medical School, Boston, USA; 4 Hospital and Health Administration, Fujita Health University Graduate School of Medicine, Toyoake, Japan; 5 Department of Health Data Science, Yokohama City University, Yokohama, Japan; 6 Faculty of Health Data Science, Juntendo University, Urayasu, Japan

**Keywords:** administrative claims data, cellulitis, chickenpox, herpes zoster, infections, NDB, seasonality, varicella

## Abstract

**Introduction:**

Cellulitis, herpes zoster, and varicella are common infectious skin diseases with significant public health implications. While these conditions have been studied in some countries, comprehensive nationwide data on their seasonality and trends in Japan remain limited. Understanding these patterns is essential for effective public health planning and disease management.

**Methods:**

This study analyzed monthly outpatient data for cellulitis, herpes zoster, and varicella using aggregated summary tables from the National Database of Health Insurance Claims of Japan for fiscal years 2014 to 2019. Seasonal patterns and trends were examined using seasonal decomposition of time series by locally estimated scatterplot smoothing.

**Results:**

Distinct seasonal patterns were observed for the three diseases. Both cellulitis and herpes zoster peaked during the summer months, while varicella exhibited a winter predominance. Trend analysis revealed a 13.5% increase in cellulitis cases over the study period. Herpes zoster cases also increased, whereas varicella cases showed a significant decline between fiscal years 2014 and 2015, coinciding with the introduction of universal varicella vaccination. The seasonal patterns were consistent with findings from previous studies in other countries.

**Conclusions:**

This nationwide study confirmed distinct seasonal patterns and trends for cellulitis, herpes zoster, and varicella in Japan. The findings enhance the understanding of the epidemiology of these infectious skin diseases at a nationwide population level and provide valuable insights for public health planning.

## Introduction

Cellulitis, herpes zoster, and varicella are common infectious skin diseases that pose a significant public health challenge globally, affecting millions of individuals annually ^(1), (2), (3)^. Understanding the seasonality and long-term trends of these conditions is important for improving public health management, optimizing resource allocation, and refining prevention strategies. Seasonal variations and trends of these diseases are often influenced by an interplay of environmental factors such as climatic conditions, human behavior, public health interventions, and individual susceptibility.

Previous studies have suggested that the incidence of these diseases exhibits distinct seasonal patterns ^(4), (5), (6)^. Despite these observations, comprehensive nationwide studies examining the seasonality and trends of these conditions in Japan remain limited. Japan, with its distinct seasonal climate, aging population, and well-established health care system, offers a unique setting to explore these epidemiological patterns. Additionally, the introduction of a universal varicella vaccination program in Japan in 2014 has likely influenced the epidemiology of varicella ^(7)^, making it essential to assess its long-term impact on disease patterns nationwide.

In this study, the seasonality and long-term trends of cellulitis, herpes zoster, and varicella in Japan were investigated using nationwide, population-based data.

## Materials and Methods

### Data source

The National Database of Health Insurance Claims and Specific Health Checkups of Japan (NDB) was used in this study ^(8)^. The NDB is a comprehensive database established by the Ministry of Health, Labour and Welfare of Japan (MHLW) that contains administrative claims data collected from all insurers across Japan. The NDB covers approximately 98%-99% of the Japanese population and includes information on patient demographics, diagnoses, procedures, and prescriptions. It has been widely used in various epidemiological studies and health services research ^(9), (10)^, including those in the infectious disease field ^(11), (12), (13)^. The secondary use of NDB data for this study was approved by the advisory committee of the MHLW. This study was approved by the ethics committee of Chiba University (project approval number M10579). Informed consent was waived due to the retrospective nature of the study and the use of anonymized data provided by the MHLW.

### Study protocol

Among the various forms of NDB data available for secondary use ^(10)^, this study utilized the aggregated summary table information, which is created by the MHLW upon specific request by the researcher. To produce the aggregated summary tables, monthly counts of patients diagnosed with cellulitis (International Statistical Classification of Diseases and Related Health Problems, Tenth Revision [ICD-10] code: L03.x), herpes zoster (ICD-10 code: B02.x), or varicella (ICD-10 code: B01.x) in outpatient settings were requested. Only clinically confirmed cases in the claims data were included (i.e., diagnosis codes marked as suspected cases were excluded). To minimize multiple counts of ongoing cases, patients with a diagnosis of cellulitis in the preceding three months were excluded. Similarly, for herpes zoster and varicella, patients with these diseases in the preceding six months were excluded.

In the aggregated summary tables, the MHLW provided the monthly number of patients for each disease between fiscal years 2014 and 2019 (i.e., from April 2014 to March 2020), stratified by sex and age group. The primary outcome of interest was the seasonal patterns and trends of cellulitis, herpes zoster, and varicella during the study period.

### Statistical analysis

To investigate the seasonality and trends of cellulitis, herpes zoster, and varicella, time series analysis was conducted. Data for each disease were first converted into time series objects with a frequency of 12 (representing monthly data) using the R package *stats*, covering between fiscal years 2014 and 2019.

Seasonal decomposition of time series by locally estimated scatterplot smoothing (LOESS) (STL) ^(14)^ was applied to decompose each time series into trend, seasonal, and remainder components. The STL method was selected for its robustness in handling non-stationary data and its flexibility in accommodating seasonal variation. The decomposition was performed using a periodic seasonal window. The R package *ggplot2* was used to visualize the time series and its components. The decomposition enabled separate analyses of the trend and seasonal components, providing insights into the underlying patterns.

Following decomposition, autocorrelation function (ACF) analysis was performed on the remainder component, which represents the residuals after removing the trend and seasonal components. The ACF analysis ensured that the remainder component did not exhibit significant autocorrelation, which would suggest that the decomposition had adequately captured the main patterns in the data.

To evaluate the significance of trends, linear regression models were fitted. Both the original time series data and the trend component extracted through STL were regressed against a time variable (a sequence from one to the length of the time series). The significance of the time coefficient in these models was used to assess the presence of a significant trend.

All statistical analyses and visualizations were conducted using R version 4.4.0 (R Foundation, Vienna, Austria).

### Assistance by artificial intelligence

The language and structure of this manuscript were refined using ChatGPT (OpenAI, San Francisco, CA, USA) and Claude (Anthropic, San Francisco, CA, USA) large language models. These models were used solely to enhance clarity and grammatical accuracy, without altering the scientific content. All artificial intelligence-suggested changes were reviewed and edited by the authors to ensure scientific integrity.

## Results

The numbers of patients diagnosed with cellulitis, herpes zoster, and varicella for the fiscal years 2014-2019 are summarized in Table 1. The data indicate a higher number of cellulitis and herpes zoster diagnoses in females compared to males, whereas varicella was more frequent among males. Notably, while the diagnosis of cellulitis and herpes zoster showed annual increases, there was a substantial decrease in the number of varicella cases between 2014 and 2015.

**Table 1.** Number of Patients with Cellulitis, Herpes Zoster and Varicella Diagnosis.

	Sex	2014	2015	2016	2017	2018	2019
Cellulitis	female	797,291	841,666	854,255	850,278	876,235	900,784
	male	672,713	712,847	717,510	722,553	745,451	768,442
	total	1,470,004	1,554,513	1,571,765	1,572,831	1,621,686	1,669,226
Herpes zoster	female	702,015	747,069	762,630	777,667	798,263	826,936
	male	486,008	518,945	531,236	542,304	558,556	576,290
	total	1,188,023	1,266,014	1,293,866	1,319,971	1,356,819	1,403,226
Varicella	female	234,327	141,941	126,371	115,438	117,514	121,828
	male	253,707	155,522	138,587	126,122	128,310	132,954
	total	488,034	297,463	264,958	241,560	245,824	254,782

**Figure 1.**
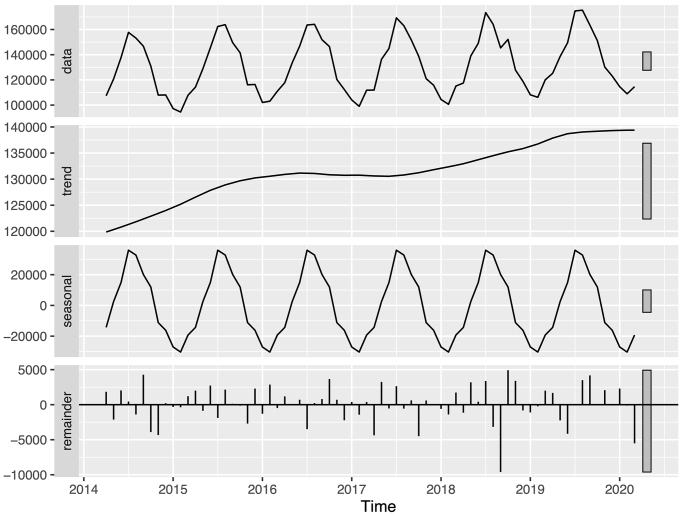
Monthly number of cellulitis diagnoses during fiscal years 2014 to 2019. The top panel shows the original time series data before seasonal decomposition of time series by locally estimated scatterplot smoothing (STL). The subsequent panels show the trend component, seasonal component, and remainder component obtained from STL analysis, respectively. STL: seasonal decomposition of time series by locally estimated scatterplot smoothing.

**Figure 2.**
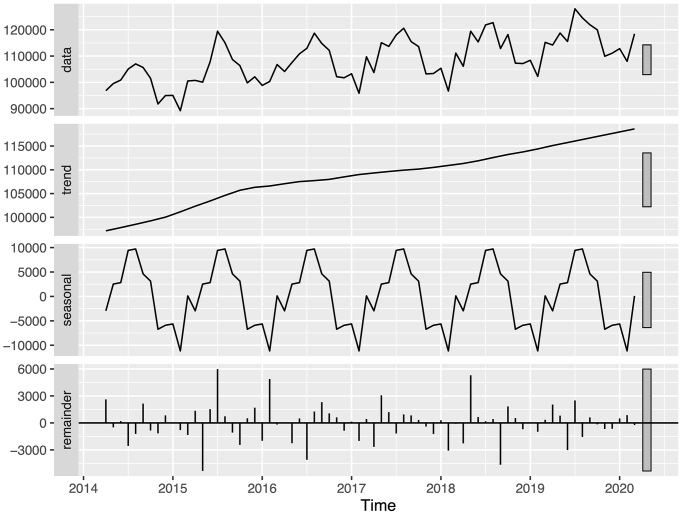
Monthly number of herpes zoster diagnoses during fiscal years 2014 to 2019. The top panel shows the original time series data before seasonal decomposition of time series by locally estimated scatterplot smoothing (STL). The subsequent panels show the trend component, seasonal component, and remainder component obtained from STL analysis, respectively. STL: seasonal decomposition of time series by locally estimated scatterplot smoothing.

**Figure 3.**
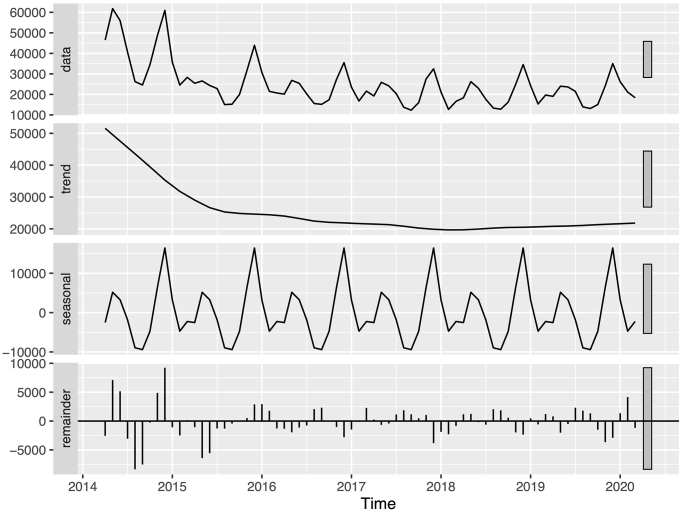
Monthly number of varicella diagnoses during fiscal years 2014 to 2019. The top panel shows the original time series data before seasonal decomposition of time series by locally estimated scatterplot smoothing (STL). The subsequent panels show the trend component, seasonal component, and remainder component obtained from STL analysis, respectively. STL: seasonal decomposition of time series by locally estimated scatterplot smoothing.

Time series analysis with STL revealed distinct seasonal patterns and trends for the three infectious skin diseases (Figure 1, 2 and 3). The remainder components, representing the unexplained variation in the data, were generally small, and the ACF analysis showed minimal autocorrelation, indicating that the trend and seasonal components captured most of the variation in the data.

Cellulitis showed a seasonal pattern with peaks in the summer months and troughs in the winter months (Figure 1). Linear regression analysis of the trend component demonstrated an increasing pattern for the study period (p < 0.001). Similarly, herpes zoster exhibited seasonal variation, with peaks occurring in the summer months (Figure 2). The trend component showed a consistent increase over the study period (p < 0.001). However, a decrease in herpes zoster diagnoses from fiscal years 2016 to 2019 for the 0-14-year age group was found (Table 2).

**Table 2.** Number of Patients with Cellulitis, Herpes Zoster and Varicella Diagnosis Stratified by Age Group and Sex.

	Age group	Sex	2014	2015	2016	2017	2018	2019
Cellulitis	0-14	Female	96,530	104,648	102,076	102,585	105,277	109,579
	0-14	Male	112,053	121,440	116,816	119,041	122,347	127,231
	15-64	Female	375,860	392,729	396,646	388,688	402,150	410,009
	15-64	Male	360,351	375,761	377,918	374,526	387,499	396,202
	≥65	Female	324,901	344,289	355,533	359,005	368,808	381,196
	≥65	Male	200,309	215,646	222,776	228,986	235,605	245,009
Herpes zoster	0-14	Female	36,224	38,927	40,815	37,377	35,445	33,900
	0-14	Male	37,950	41,519	43,277	40,111	38,364	36,488
	15-64	Female	329,425	345,758	353,147	361,241	370,958	386,593
	15-64	Male	242,315	256,234	261,881	268,773	278,796	289,514
	≥65	Female	336,366	362,384	368,668	379,049	391,860	406,443
	≥65	Male	205,743	221,192	226,078	233,420	241,396	250,288
Varicella	0-14	Female	225,539	134,549	119,429	108,383	109,908	113,548
	0-14	Male	245,306	148,644	132,033	119,279	120,677	124,635
	15-64	Female	8,176	6,784	6,447	6,546	6,995	7,632
	15-64	Male	7,793	6,428	6,032	6,368	7,088	7,747
	≥65	Female	612	608	495	509	611	648
	≥65	Male	608	450	522	475	545	572

Conversely, varicella displayed a distinct seasonal pattern with peaks in the winter months (Figure 3). The trend component showed a substantial decrease between fiscal years 2014 and 2015, followed by a gradual decline (p < 0.001). A slight increase was observed in fiscal year 2019.

## Discussion

In this study, the seasonality and trends of three common infectious skin diseases in Japan were investigated using nationwide claims data. Distinct seasonal patterns were found, with cellulitis and herpes zoster peaking in the summer months, while varicella peaked in the winter. The trend analysis revealed increasing patterns for both cellulitis and herpes zoster over the study period. In contrast, varicella showed a marked decrease between fiscal years 2014 and 2015.

The seasonal patterns observed in this Japanese nationwide data align with findings from previous reports ^(5), (15), (16)^. For example, previous research using nationwide inpatient data in the USA showed that hospital admissions for cellulitis are strongly associated with warmer weather ^(15)^. Additionally, a study from Western Australia showed that presentations of lower leg cellulitis in non-tropical regions were higher in summer than in other seasons, whereas no seasonality was observed in warmer tropical regions ^(5)^. Furthermore, a study based on Google Trends data in 31 countries showed seasonal variation in internet search volume for cellulitis, with peaks in the summer ^(16)^. This pattern of public interest, consistent with clinical data, provides additional evidence of cellulitis seasonality on a global scale. The seasonality of cellulitis may be attributed to warmer climate affecting pathophysiological factors of the host, as well as social and environmental factors such as increased outdoor activities and exposure to environmental pathogens during warmer months.

The number of patients with cellulitis increased by 13.5% between fiscal years 2014 and 2019. Although trends in outpatient cellulitis cases are not well documented, a study in the USA showed that hospitalizations for cellulitis approximately doubled from 1998 to 2013, resulting in a substantial increase in health care costs ^(17)^. The upward trend in this study underscores the increasing burden of cellulitis on Japanese health care systems and highlights the need for effective preventative strategies. Raising public awareness about cellulitis risk factors, including seasonality, may aid in reducing incidence and associated health care costs ^(18)^.

Interestingly, a higher number of cellulitis diagnoses in females compared to males was found throughout the study period, which contrasts with several previous hospital-based studies that reported a male predominance in cellulitis cases ^(19), (20)^. This discrepancy might be explained by differences in health care settings (outpatient vs. inpatient), as this study captures milder cases that do not require hospitalization. Additionally, women generally utilize outpatient health care services more frequently and at earlier stages of disease than men ^(21), (22)^, which may lead to higher diagnosis rates in ambulatory settings. Furthermore, the nationwide database provides a more comprehensive picture of cellulitis epidemiology across Japan, whereas hospital-based studies may have selection biases that influence gender distribution. Determining the precise reasons for these gender differences would require specifically designed studies that include detailed risk factor analysis across both outpatient and inpatient settings, which is beyond the scope of the current research. Nevertheless, these findings highlight the importance of considering health care setting when interpreting epidemiological data on infectious skin diseases.

In this study, the seasonal pattern of herpes zoster showed peaks in the summer months, which contrasts with the winter predominance of varicella, despite both diseases being caused by the varicella-zoster virus (VZV) ^(3)^. Previously, a 10-year survey in Miyazaki, a prefecture in Japan, showed the same seasonal patterns for herpes zoster and varicella between 1996 and 2006. Another study using administrative claims data in the USA showed a peak of herpes zoster in the summer ^(23)^. While varicella transmission is known to be facilitated by close contact, especially among children in childcare centers and schools during winter months ^(3)^, a study suggested the involvement of ultraviolet (UV) effects on the immune system based on a strong correlation between UV radiation levels and herpes zoster reactivation ^(24)^.

Herpes zoster also showed an increasing trend throughout the study period, consistent with previous studies reporting a global increase in herpes zoster incidence ^(2)^. While multiple factors might contribute to this trend, including aging populations, some researchers have hypothesized that decreased exposure to varicella cases (e.g., due to the introduction of vaccination) might reduce VZV-specific immunity in the population, potentially leading to increased herpes zoster incidence ^(2), (25)^. However, a long-term study in the US showed that herpes zoster incidence increased more than fourfold over a 60-year period (1945-2007). The rate of increase before and after the introduction of the varicella vaccination program was comparable, suggesting that other factors are likely responsible for this long-term increasing trend ^(26)^. Notably, this study also found a decline in herpes zoster among children aged <10 years following the introduction of varicella vaccination, which aligns with findings in this study.

In Japan, it is worth noting that varicella infection was largely uncontrolled, with vaccine coverage estimated at only 40% prior to the introduction of universal immunization in November 2014 ^(7)^. Thus, the substantial decrease in varicella cases observed between fiscal years 2014 and 2015 in this study reflects the impact of this universal immunization program ^(27), (28), (29)^. It is also noted that only the live VZV vaccine, which could not be used for immunocompromised patients at high risk for herpes zoster, was available in Japan during most of the study period. The introduction of recombinant zoster vaccine (Shingrix^Ⓡ^) ^(30), (31)^ in 2020 provided an alternative option for this vulnerable population in Japan ^(32), (33), (34)^, suggesting that the prevalence of herpes zoster may also decrease in the future.

The strength of this study was the use of NDB, which covers approximately 98%-99% of the Japanese population, offering comprehensive nationwide data for analysis ^(8)^. However, several limitations of this study should be noted. First, as the aggregated summary tables from the NDB provided by the MHLW were used, detailed patient-level information such as comorbidities, severity of disease, and specific treatment patterns were not available. Second, the diagnostic accuracy of the diagnosis could not be validated in this study. However, these limitations would not substantially affect the analysis of seasonality and temporal trends at the population level, as demonstrated by findings that align with previous studies using more detailed data collection methods.

In conclusion, this nationwide study confirmed distinct seasonal patterns and trends of cellulitis, herpes zoster, and varicella in Japan. The findings regarding seasonal variations and long-term trends align with previous reports from Japan and other countries. Furthermore, the seasonal patterns and trends identified in this study may help inform public health strategies and resource allocation for these common infectious skin diseases.

## Article Information

### Conflicts of Interest

None

### Sources of Funding

This work was supported by JSPS KAKENHI Grant Number JP21K08294.

### Acknowledgement

Hideaki Miyachi was supported by fellowships for studying abroad from the International Medical Research Foundation, the Japanese Dermatological Association, and JSPS Overseas Research Fellowships. The funding organizations played no role in the study design, data collection or analysis, preparation, or approval of the manuscript, or the decision to submit the manuscript for publication.

### Author Contributions

Conceptualization: Hideaki Miyachi and Daisuke Sato; Methodology: Hideaki Miyachi, Daisuke Sato, and Sayuri Shimizu; Data analysis: Hideaki Miyachi; Data interpretation: Daisuke Sato, Sayuri Shimizu, Yaei Togawa, Kentaro Sakamaki, and Kensuke Yoshimura; Data curation: Hideaki Miyachi, Daisuke Sato, and Kensuke Yoshimura; Writing - Original Draft: Hideaki Miyachi; Writing - Review & Editing: Daisuke Sato, Sayuri Shimizu, Yaei Togawa, Kentaro Sakamaki, and Kensuke Yoshimura; Supervision: Daisuke Sato and Kensuke Yoshimura; Funding acquisition: Hideaki Miyachi. Hideaki Miyachi and Daisuke Sato contributed equally to this work.

### Approval by Institutional Review Board (IRB)

This study was approved by the ethics committee of Chiba University (project approval number M10579). Informed consent was waived due to the retrospective nature of the study and the use of anonymized data provided by MHLW for this study.
